# Field Cricket (*Gryllus bimaculatus*) and Spirulina (*Arthrospira platensis*) Powders as Environmentally Friendly Protein Enrichment Ingredients in Corn Snacks

**DOI:** 10.3390/foods13152390

**Published:** 2024-07-28

**Authors:** Millena Ruszkowska, Małgorzata Tańska, Joanna Miedzianka, Przemysław Łukasz Kowalczewski

**Affiliations:** 1 Department of Quality Management, Faculty of Management and Quality Science, Gdynia Maritime University, 81-225 Gdynia, Poland; 2 Department of Food Plant Chemistry and Processing, University of Warmia and Mazury in Olsztyn, 10-726 Olsztyn, Poland; m.tanska@uwm.edu.pl; 3 Department of Food Storage and Technology, Wroclaw University of Environmental and Life Sciences, 51-630 Wrocław, Poland; joanna.miedzianka@upwr.edu.pl; 4 Department of Food Technology of Plant Origin, Poznań University of Life Sciences, 60-624 Poznań, Poland

**Keywords:** colour, edible insects, extrusion, nutritional value, sensory acceptance, texture, water sorption

## Abstract

Unconventional protein sources are currently extensively studied as food ingredients. This study aimed to evaluate the effect of 1.5% and 3% field cricket powder (GB) and 2–8% of its mixture (1:1) with spirulina powder (S) on the nutritional value, physicochemical properties, and sensory characteristics of corn extrudates. Additionally, 2% baking powder (BP) was added to assess its impact on the properties of the enriched extrudates. The results showed that both GB and GB + S improved nutritional value, with protein content increasing by up to 46% and higher levels of essential amino acids, particularly leucine and valine. However, these ingredients decreased the expansion ratio (by up to 15%), colour lightness (by up to 30%), and yellowness (by up to 47%) and increased the hardness (by up to 25%) of the corn extrudates. The S addition positively influenced product storage stability but decreased its sensory acceptance, especially aroma and taste. The BP addition mitigated the negative effects of higher GB and GB + S concentrations, particularly on sensory characteristics. In conclusion, incorporating up to 6% of the GB + S mixture provides a higher protein content with only minor changes to the product’s characteristics compared to GB. Ratings exceeding 4.2 points indicate the good acceptability of these snacks.

## 1. Introduction

Edible insects and algae (especially spirulina) are increasingly recognised as unconventional sources of protein and have become the focus of extensive scientific research [1,2,3,4,5,6]. Literature data show that insects are highly valuable in terms of nutritional value [7], containing substantial amounts of protein, fat, vitamins, and minerals. However, the protein content of insects varies significantly depending on the species, developmental stage, and rearing method (whether through farming or wild trapping), with levels ranging from 13% to over 77% of dry matter [8]. Additionally, researchers indicate that factors such as country of origin, environmental conditions, and thermal treatment methods (cooking, baking, or roasting) significantly impact the nutritional value of edible insects [9,10]. For example, during the heat treatment process, proteins may be denatured, and amino acids could be destroyed. The digestibility of the protein may be improved or impaired depending on how the protein is transformed during processing and its interactions with other phytochemicals [11]. Available data also suggest that insect consumption can positively affect human health. Stull et al. [12] reported that insect consumption can stimulate the growth of intestinal microflora and reduce blood levels of tumour necrosis factor α (TNF-α). Moreover, chitin and chitosan, substances present in crickets, have been shown to inhibit pathogenic microorganisms in the gut [13]. Zielińska et al. [14] also observed, in in vitro studies, that insects have beneficial properties for the prevention and treatment of metabolic syndrome. They reported that insects such as *Gryllodes sigillatus*, *Tenebrio molitor*, and *Schistocerca gregaria* are good sources of bioactive peptides with inhibitory activity against selected enzymes that may be involved in the pathogenesis of metabolic syndrome. In contrast, spirulina is considered an excellent choice for enhancing the nutritional value of cereal products, as it contains a high protein content (typically between 60% and 70%) with high digestibility and a valuable amino acid profile close to that recommended by the FAO [15]. It is also characterised by a low content of nucleic acids. Spirulina’s protein contains sufficient amounts of essential amino acids, except for methionine, cysteine, and lysine. Furthermore, spirulina is rich in other bioactive compounds such as carotenoids, chlorophylls, phycocyanin, vitamins (B_1_, B_2_, B_3_, B_12_), minerals (iron, copper, magnesium), and unsaturated fatty acids, including γ-linolenic and linoleic acids [16,17,18]. However, Siva Kiran et al. [19] reported that based on the composition and Recommended Dietary Allowance (RDA) values, it is recommended for healthy adults to consume less than 4 g of spirulina per day due to the presence of excess vitamin A, and not more than 10 g per day, as it exceeds the RDA values for heavy metals.

Despite the high potential of insects as an unconventional protein source, their consumption in whole form faces significant barriers, primarily due to low acceptance among Western societies [10,20,21,22,23]. This reluctance is mainly attributed to food neophobia, which is driven by factors such as disgust and repulsion [24,25,26]. In contrast, the introduction of algae into food products encounters several obstacles. Consumer attitudes towards the sensory characteristics of many algal species—such as fishy taste, smell, and unusual colour—pose significant challenges [27]. Additionally, the high cost of harvesting and processing algae is a crucial factor. Legal issues and necessary testing for product approval further hinder the use of both algae and insects in food production [28,29]. While barriers related to production costs and regulatory approvals can be addressed relatively quickly, achieving consumer acceptance for products containing insects and algae may be a prolonged process. Researchers suggest that consumer acceptance and willingness to try new insect-containing products increase when these are incorporated in a familiar, convenient, and masked form, such as powder [25,30]. For algae, the low amount of this ingredient or the use of additional processes like encapsulation to mask the fishy taste and smell may be crucial for improving product acceptance [29].

Extrusion is recognised as an encapsulation technique that can effectively mask undesirable flavours and tastes in food products. During the extrusion process, materials are subjected to high pressure and temperature as they are forced through a die, leading to significant physical and chemical transformations. This process can encapsulate bioactive compounds, flavours, and nutrients within a starch or protein matrix, thereby protecting them from degradation and controlling their release [31]. Since extrusion effectively masks off-flavours, this makes it a valuable method for incorporating unconventional ingredients with specific sensory characteristics, such as edible insects and spirulina, into mainstream products [21,32]. There have been limited studies on incorporating insects into extruded products. For example, Ribeiro et al. [21] and Igual et al. [33] added up to 15% house cricket (*Acheta domesticus*) powder to corn grits to produce snacks, Azzollini et al. [34] obtained wheat snacks with 10% and 20% mealworm larvae (*T. molitor*) powder, García-Segovia et al. [35] investigated the physicochemical and nutritional properties of corn snacks with 5% *Alphitobius diaperinus* and *T. molitor* powders, and Alam et al. [36] produced pellets with fly larvae (*Hermetia illucens*) powder, both alone and mixed with corn flour in various ratios (1:1 to 1:3). Additionally, Smarzyński et al. [37] showed that cricket powder could enhance consumer acceptance of meat products, using pork pâté as an example. Smetana et al. [38] replaced 15–40% of soy protein concentrates with insect protein concentrates from *A. diaperinus* and *T. molitor* in meat analogues. Similarly, Kiiru et al. [39] produced meat analogues incorporating 15–45% whole or partially defatted *A. domesticus* powder. While these cited studies indicate a significant increase in the nutritional value of extruded products after the incorporation of edible insect powder, they also highlight a reduction in the expansion ratio and a deterioration in sensory characteristics when high percentages of this ingredient are used in the formulation. The addition of 2–10% was found to be acceptable, depending on the product. Also, some scientific studies exploring algae to extruded products, e.g., Morsy et al. [40], Lucas et al. [41], and Tańska et al. [42], investigated the incorporation of spirulina into corn snacks, while Uribe-Wandurraga et al. [43] obtained corn extrudates enriched with *Arthrospira platensis*, *Chlorella vulgaris*, and *Nannochloropsis gaditana* biomass. However, acceptable levels of these additives in extrudates were found to be lower than those for edible insects, up to 8%, when an additional 2% leavening agent (baking powder) was introduced [42].

*Gryllus bimaculatus* (field cricket) is a less studied protein source compared to *A. domesticus* (house cricket), especially in the context of extruded food products. To date, Nam et al. [44] evaluated the potential use of various fractions of *G. bimaculatus* mixed with soybean protein isolate to form gels as a meat analogue. Loypimai et al. [45] reported high protein contents (59–70%) in powders obtained from both crickets, but *G. bimaculatus* powder exhibits significantly higher levels of B-group vitamins (B_1_, B_2_, B_5_, B_6_, B_12_), vitamin A, and β-carotene, and twice as much as *cis*-9-oleic acid compared to *A. domesticus* powder. To the best of our knowledge, no studies have examined the impact of the mixture of this edible insect and spirulina on the properties of corn snacks. The research team hypothesised that incorporating a mixture of low amounts of insect and spirulina powders into corn extrudates would improve their nutritional value without significantly altering their expansion, physicochemical properties, and sensory quality. This was verified by comparing the effect of field cricket powder and its mixture with spirulina powder additions on the nutritional value (content of main chemical compounds and amino acids), physicochemical properties (expansion ratio, bulk density, hardness, colour parameters, functional water properties, and sorption parameters), and sensory characteristics (appearance, colour, aroma, taste, and crispness) of corn extrudates. Additionally, the effect of the addition of baking powder was assessed to check its improving potential on the physical properties of the enriched extrudates, as demonstrated in a previous study [42].

## 2. Materials and Methods

### 2.1. Research Materials

The corn extrudate research materials were produced with added protein from unconventional sources (commercial powders of edible insects and spirulina). The products were made from prepared blends obtained from the corn grits with a granulation of 0.36–0.65 mm purchased from Sante (Sobolewo, Poland), baking powder (BP) from Dr. Oetker (Bielefeld, Germany), field cricket powder (GB) from JR Unique Foods (Udon Thani, Thailand), and spirulina powder (S) from Bio Planet (Leszno, Poland). All raw materials were purchased from local supermarkets and were characterised by their current shelf life, appropriate organoleptic properties, and typical moisture content (12.45% for corn grits, 8.64% for field cricket powder, 10.22% for spirulina powder, and 3.68% for baking powder). Until the extrusion process, they were stored in a dry, cool, and dark place. Table 1 shows the nutritional values of these raw materials as declared by manufacturers. The initial composition of the blends was derived from Tańska et al. [42] and Ruszkowska et al. [46]. However, the final proportions in the product formulation were established based on preliminary results from extrusion tests.

### 2.2. Preparation of Extrudates

The blends produced (each of 0.5 kg) were subjected to an extrusion process, which was carried out using a single-screw extruder type S45A-12-10U from Metalchem (Gliwice, Poland) with a cylinder (length 12.0 mm, nominal diameter 15.0 mm) connected to the discharge nozzle (diameter 4.5 mm). A screw speed of 125 rpm (rotations per minute), feed rate of 25 kg/h, and temperatures of 105 °C, 130 °C, and 110 °C (zone I, zone II, and head, respectively) were applied. The process produced extrudates with different ingredient contents and a control product (R), an extrudate without insect and spirulina powders (Table 2). The cooled extrudates (4 h at 19 ± 2 °C and a relative humidity of 54 ± 2%) were packed in airtight polyethylene bags and stored in a cool, dry place for the analyses described in the test methodology.

### 2.3. Evaluation of Basic Composition

The nutrient content of the extrudates produced was determined using AOAC methods [47]: protein content (AOAC 990.03), fat content (AOAC 920.39), ash content (AOAC 942.05), and total dietary fibre content (AOAC 962.09). Carbohydrate content was defined as the rest of the dry matter at 100%. Total energy was calculated using conversion factors of 4 kcal/g for carbohydrates, 4 kcal/g for protein, and 9 kcal/g for fat.

### 2.4. Evaluation of Amino Acid Profile and Calculation of Amino Acid Score

The extrudates were subjected to acid hydrolysis [48] and placed in an automatic amino acid analyser AAA400 (INGOS, Prague, Czech Republic). A two-coordinate photometer (440 and 570 nm) was used for the detector. The dimensions of the column filled with an ion exchanger (Ostion LG ANB, INGOS, Prague, Czech Republic) were 350 × 3.7 mm, while the column temperature was maintained between 40 and 70 °C and the detector temperature was 121 °C. Amino acids were quantified using the ninhydrin method. Glutamine and aspartic acid were expressed as glutamic acid and aspartic acid, respectively. No analysis was performed for tryptophan. Calculations were performed using the CHROMuLAN computer programme v. 0.60.3 (PiKRON, Prague, Czech Republic). The amino acid score (AAS) value was calculated for adults using the standard method recommended by FAO/WHO [15]:

$$AAS=\frac{content\;of\;exogenous\;amino\;acids\;in\;the\;sample}{recommended\;content\;of\;exogenous\;amino\;acids}(\%)$$

### 2.5. Analysis of Functional Water Properties

The water content of the products was determined by thermal drying to a constant weight at 105 °C at normal pressure [42]. Water activity (a_w_) was measured using an AquaLab 4TE instrument (AS42.14.0, Decagon Devices, Inc., Pullman, WA, USA) with an accuracy of ±0.0003 at 20 ± 2.5 °C. 

The water absorption index (WAI) and water solubility index (WSI) were determined following the procedure described by Sharma et al. [49]. Approximately 1.5 g of ground extrudate sample was used for each determination. The samples were mixed with 15 mL of distilled water and shaken. The resulting solutions were centrifuged at 3000× *g* for 15 min. The resulting liquid was transferred to glass petri dishes and dried in an oven at 110 °C. WAI (g/g) was calculated as the ratio of the weight of the gel after removing excess water to the dry weight of the sample, while WSI (%) represented the weight of dry matter in the supernatant as a percentage of the original sample weight.

### 2.6. Calculation of Expansion Ratio

The expansion ratio (ER, or expansion ratio) of the extrudates was calculated from the ratio of the diameter of the extrudate to the diameter of the extruder die [50].

### 2.7. Evaluation of Bulk Density

The specific density (BD, or bulk density) of the extrudates was determined by the mass-to-volume ratio of the individual extrudates [50].

### 2.8. Measurement of Mechanical Properties

Mechanical properties were determined using a uniaxial compression test (model 4301, Instron Corp., Canton, MA, USA). Extrudates were compressed at a constant rate of 50 mm/min, and the maximum force (hardness) was recorded up to an assumed 50% strain. The compressive strain was estimated as the force divided by the cross-sectional area of the extrudates [51].

### 2.9. Measurement of Colour Parameters

The colour of the extrudate cross-sections was measured using a digital image analysis kit and expressed in the parameters of CIE L*a*b* model [46]. Fifteen colour measurements were taken each time. Cross-sectional images were captured using a Nikon DXM-1200 camera (Nikon Inc., Melville, NY, USA). Colour parameters were determined using LUCIA G v. 4.8 software (Laboratory Imaging, Prague, Czech Republic). The light source was a set of Kaiser RB 5004 HF high-frequency daylight fluorescent lamps with 4 bulbs with a colour temperature of 5400 K (Kaiser Fototechnik GmbH and Co. KG, Buchen, Germany). The total colour difference (ΔE) was calculated using the following formula:

$$\Delta E=\sqrt{{\Delta L}^{*2}+{\Delta a}^{*2}+{\Delta b}^{*2}}$$

where: ΔL*, Δa*, Δb*—the differences between the values of the individual parameters of product R (control sample) and the products with the introduced ingredients. 

### 2.10. Evaluation of Sorption Properties 

The storage stability of the produced snack products was investigated based on their sorption characteristics using water vapour sorption isotherms. The extrudates were placed in a hygrostat with a water activity value (a_w_) range from 0.07 to 0.98 for 45 days at 25 °C. Mathematical models of the sorption isotherms were developed using the BET equation (Brunauer, Emmett, and Teller) within the range of a_w_ = 0.07–0.33. Parameters, such as the monomolecular layer capacity of the extrudates, sorption surface area, and energy constant, were determined [52]. The analysis of results was conducted using TableCurve 2D v. 5.01 (Systat Software Inc., Palo Alto, CA, USA) software. The fit of the empirical data to the BET equation was characterised using the coefficient of determination (R^2^), the standard error estimate (Fit-StdErr), and the Fstat.

### 2.11. Sensory Evaluation

**Figure 1:**
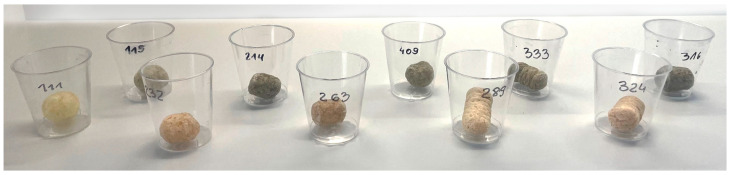
Presentation of extrudate samples prepared for sensory evaluation. Code numbers were used to code the analyzed samples (111 for R, 115 for 1 GB + 1 S, 214 for 2 GB + 2 S, 409 for 3 GB + 3 S, 333 for 4 GB + 4 S + BP, 316 for 4 GB + 4 S, 232 for 1.5 GB, 263 for 3 GB, 289 for 1.5 GB + 2 BP, and 324 for 3 GB + 2 BP).

The sensory evaluation of the extrudates was carried out in the Sensory Evaluation Laboratory using a 5-point scale, where 1 point represented the lowest level of acceptability, and 5 points indicated the highest. Our study and the survey protocol received approval from the Rector’s Committee for the Ethics of Scientific Research Involving Humans at Poznań University of Life Sciences (Resolution No. 1/2023; 7 March 2023). A panel of 20 trained individuals evaluated five sensory attributes: appearance, colour, aroma, taste, and crispness, and also provided an overall desirability rating for the products. All panellists had previous experience in sensory evaluation, and various screening tests were employed to ensure their suitability according to ISO 8586 [53] (e.g., assessing impairment, sensory acuity, and ability to describe and communicate sensory perceptions). They were familiarised with the samples and typical sensory attributes of extruded products and the raw materials used during two 90 min training sessions. During the testing session, samples were presented in a random order to the panellists at 20 °C under normal lighting conditions. The samples were placed in containers labelled with a 3-digit code (Figure 1). Each panellist received an evaluation sheet and a pen, as well as a cup of water to cleanse their palate before evaluating the next sample.

### 2.12. Statistical Analysis

Two independent production runs, each conducted in triplicate (n = 6), were used for the analyses unless otherwise specified. The obtained results were analysed using Statistica v. 13.3 software (Tibco Software, Palo Alto, CA, USA). Verification of all hypotheses was performed at a significance level of α = 0.05, based on the probability value (*p*-value) of the test. A statistically significant difference in the dependent variable was assumed when *p ≤* 0.05.

## 3. Results and Discussion

### 3.1. Nutritional Value of Extrudates

The basic nutrient contents presented in Table 3 show that the addition of cricket powder and spirulina powder significantly increased the protein content of the analysed extrudates. As the proportion of corn grits was replaced with cricket and spirulina powders, the observed changes became more pronounced. Similar trends were noted for dietary fibre, fat, and minerals (as indicated by ash content). Notably, the proportion of carbohydrates decreased. Extruded corn snacks are not typically regarded as wholesome despite being the most commonly consumed snacks. Consequently, extensive research has focused on enriching corn snacks with new ingredients to enhance their nutritional value or confer health-promoting properties [54]. Thus, it can be concluded that both ingredients, GB and S, enhanced the nutritional value of the snacks. 

Among products enriched with cricket powder, the highest protein, fibre, fat, and ash contents were found in the extrudates with a 3% addition of insect powder (samples 3 GB + 2 BP and 3 GB). However, the incorporation of both cricket and spirulina powders into the extrudate formulations significantly improved the nutritional value of the produced extrudates. Based on the conducted research, the product containing the highest 4% addition of cricket powder and 4% spirulina (sample 4 GB + 4 S) exhibited the most favourable nutritional value among all the produced extruded product variants. Despite the high protein content in enriching raw materials such as cricket powder and spirulina (Table 1), the final product with the highest percentages of these components (sample 4 GB + 4 S) achieved a protein content of 11.81% (compared to a theoretical content of 12.92% in the raw material mixture). This is likely due to the extrusion process conditions, particularly the temperature range used, which may have significantly influenced the nutritional value of the produced extrudates. It is important to note that the studied species of insects, *G. sigillatus*, *T. molitor*, and *S. gregaria,* are good sources of bioactive peptides with in vitro inhibitory activity against selected enzymes that may be involved in the pathogenesis of the metabolic syndrome. Generally, the heat treatment process has a substantial impact on the improvement of these properties [13].

Higher protein content is indeed a beneficial aspect, but it is not the only factor; the change in the amino acid profile of the corn-based product is also key to assessing its nutritional value [47,48]. Based on the analysis of the amino acid profile of extrudates made from corn grits enriched with field cricket powder and spirulina, it can be concluded that samples containing both ingredients showed the highest total amino acid content (76.58−95.70 mg/g) compared to the control sample (73.71 mg/g). Extrudates enriched with cricket and spirulina powders contained higher levels of total essential amino acids, ranging from 32.76 to 39.01 mg/g compared to the control sample (29.05 mg/g), which is also reflected in the total protein content of these samples (Table 4). In contrast, products obtained with only cricket powder showed higher levels of total essential amino acids (31.35−33.55 mg/g). Regardless of the protein source used, leucine was the predominant essential amino acid in the analysed enriched extrudates (8.83−12.40 mg/g). In all samples, glutamic acid was the predominant endogenous amino acid, with its content ranging from 12.51 mg/g (control sample) to 18.89 mg/g (sample 4 GB + 4 S). It was found that enriching the corn extrudates with as little as 1.5% field cricket powder increased the content of all amino acids, thus enhancing the nutritional value of the finished product compared to the control sample. 

While alternative protein sources such as pea protein, soy protein, and quinoa have been extensively studied and utilised in extruded products [32,55,56,57], cricket powder and spirulina offer unique nutritional and environmental advantages. Combining the protein-rich cricket powder with nutrient-dense spirulina creates a product with balanced amino acids, making them standout options for enriching snack foods.

### 3.2. Water Functional Properties of Extrudates

The results of the water content and activity analysis in the tested extrudates are presented in Table 5. The lowest water content was observed in the extrudates containing both the highest contents of protein sources and baking powder (sample 4 GB + 4 S + 2 BP), while lower amounts of cricket and spirulina powders (2% and 3%) significantly increased the moisture content of the extrudates. The water content of extrudates is determined by both the chemical composition and moisture content of the mixture prepared for extrusion and the parameters used during the process [56]. Since the moisture content of the mixtures was standardised and the process parameters were unchanged, it can be assumed that the interactions between the ingredients resulted in stronger binding and retention of water, which evaporated to a lesser extent after the extrusion process. Additionally, the use of baking powder affected the properties of the biopolymers and reduced the water content of the extrudates. It was found that higher water content highly correlated with higher water activity in the tested products (calculated correlation coefficient (r) was 0.75). Water content and activity significantly impact the characteristics of corn extrudates, including their texture, taste, shelf life, and even sensory quality. High water content can lead to a loss of crunchiness, a crucial characteristic of these types of products, making the snacks rubbery and less appealing to consumers [58]. Conversely, low water activity helps maintain freshness and crunchiness for longer periods of time, preventing the growth of microorganisms and enzymes responsible for food spoilage [59]. Furthermore, as indicated by Makowska et al. [60], lower water activity also reduces the rate of chemical and biochemical reactions in the extrudates, which can deteriorate their nutritional or sensory properties.

No effect of cricket powder addition on the water absorption of the extrudates was observed, whereas the addition of baking powder resulted in a statistically significant increase (*p* ≤ 0.05) in water absorption index (WAI) values. Literature data indicate that cricket powders exhibit low water absorption [61], while baking powder increases the hydrophilicity of proteins and their solubility [62,63]. Technological processing of insects, particularly high-temperature roasting and drying, denatures the protein fraction, leading to reduced water absorption. In contrast, the use of spirulina powder enhances the water-binding capacity of extrudates, with no additional beneficial effect observed from baking powder. 

The WAI depends on both the composition and the temperature of the extrusion process; higher extrusion temperatures improve starch gelatinisation and reduce dextrin formation. Process parameters such as intensive mechanical shear, high pressure, and temperature also influence water solubility index (WSI) values [64,65,66,67]. Since all extrudates were produced using the same extrusion process parameters, the observed changes in WAI and WSI values can be attributed to the modification of raw materials, as confirmed by other studies [65,68]. The product formulation significantly altered the WSI, with higher WSI values observed when the basic compound (corn grits) was replaced with insect and spirulina powders. This may be attributed to the lower moisture content of the samples subjected to the extrusion process. Morsy et al. [40] explained that the decrease in WSI with increasing moisture content is due to the higher blend moisture resulting in lower viscosity of the mass liquefied in the extruder. Consequently, the intensity of the shearing forces on the processed material was reduced, which also decreased the degree of dextrinisation of the starch polymers. However, a high WSI value is undesirable from a nutritional point of view, as it indicates rapid digestion and intestinal absorption [69].

### 3.3. Expansion Properties and Hardness of Extrudates

**Figure 2:**
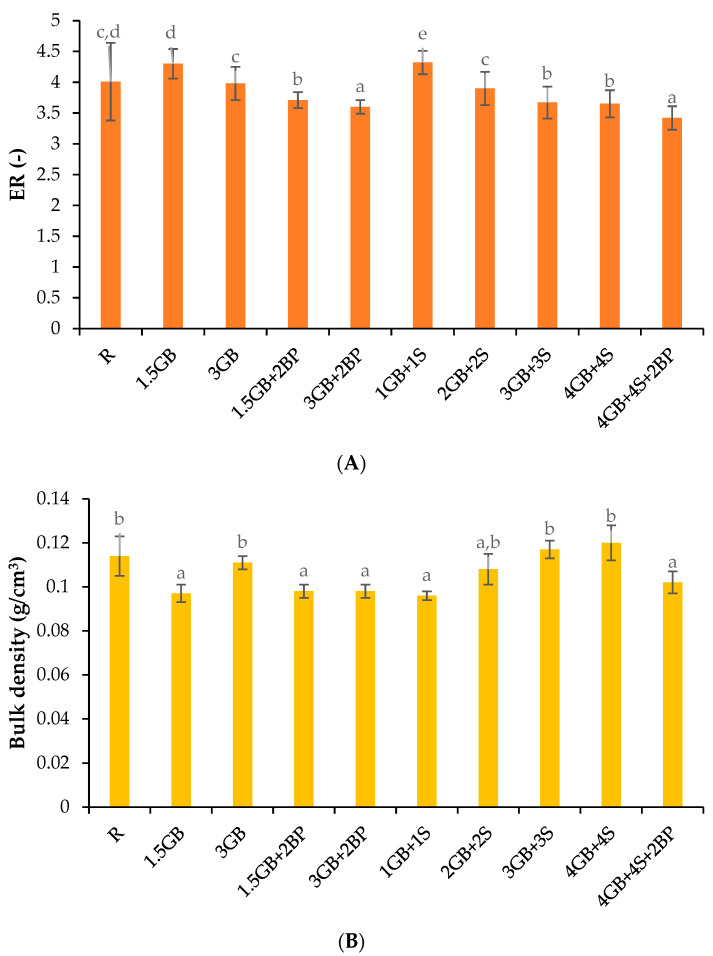

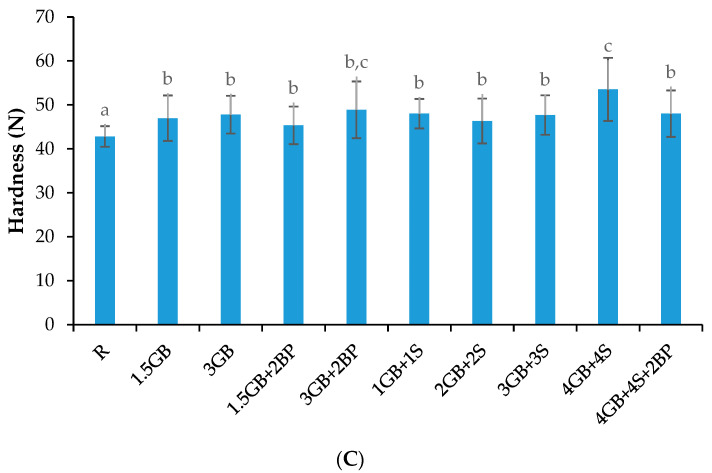
Expansion ratio (ER) (**A**), bulk density (**B**), and hardness (**C**) of control and enriched extrudates. Mean values with the same letters were not significantly different (α = 0.05). See Table 2 for explanation of sample codes.

Modifying the chemical composition of the enriched extrudates resulted in changes to their expansion properties, as shown in Figure 2. The use of the lowest level of added cricket powder or a mixture of this powder with spirulina powder (samples 1.5 GB and 1 GB + 1 S) increased the ER. However, further increases in their content negatively affected the expansion process, resulting in a reduction of ER by up to 15% when the largest amounts of both powders and leavening agent (sample 4 GB + 4 S + 2 BP) were used (Figure 2A). Robin et al. [70] indicate that a high dietary fibre content in the extruded raw material results in a decrease in the expansion ratio and an increase in the density of the extrudates obtained. The expansion properties of extrudates, such as expansion ratio (ER) and bulk density (BD), influence final consumer acceptance and depend on the chemical composition of the extruded blend [58]. It is worth mentioning that a higher ER generally leads to better consumer perception of the extrudates. Both insect powder [10] and spirulina powder [71] contain higher dietary fibre contents than corn grits (Table 1), which contributed to the lower ER values. The expansion ratio may also be influenced by the increased fat content introduced with field cricket powder (Table 1). Ilo et al. [72] reported that the addition of small quantities of fat (<3%) during the extrusion process has minimal impact on expansion, whereas amounts exceeding 5% lead to significant decreases in extrudate expansion. Yovchev et al. [73] explained this phenomenon by attributing it to the lubricating effect of fat, which prevents starch molecules from undergoing breakdown under shear forces, thereby reducing the degree of starch conversion and negatively affecting expansion. Conversely, proteins can have either a positive or negative impact on expansion due to their ability to alter water distribution within the matrix, depending on the type and concentration of protein [74].

In extruded products, texture is of major importance, with hardness and crispness often being desirable attributes. The hardness of the extrudates obtained was found to vary from 42.80 to 53.52 N (Figure 2C). A significant increase in hardness was observed with increasing contents of cricket powder or a mixture of both protein sources. The use of baking powder significantly reduced hardness for extrudates with a mixture of cricket and spirulina powders and for extrudates with 1.5% insect powder. The increase in hardness with higher insect powder addition may be due to the higher fat content compared to corn grits (18.0% vs. 1.7%, Table 1). Similar findings were reported by Igual et al. [31] for corn snacks with different quantities of house cricket powder and by Azzollini et al. [32] for snacks produced from wheat flour and ground yellow mealworm larvae. 

In the present study, a high positive correlation was observed between extrudate protein content and hardness (calculated r = 0.70). The increased hardness of protein-enriched extrudates may be associated with reduced expansion (Figure 2A) and thicker cell walls (Figure 2C), as thinner cell walls tend to fracture more easily [75]. Reduced expansion is directly related to increased hardness and crispness because proteins interfere with water distribution in the matrix due to their conformation and hygroscopic properties, making it difficult for starch to absorb water during cooking [76]. Van der Sman et al. [77] suggested that bubble expansion can be enhanced by inorganic leavening agents such as sodium bicarbonate (a component of baking powder). However, the beneficial effect of this additive was not observed in corn extrudates containing the highest amount of cricket powder (sample 3 GB + 2 BP), indicating the complex processes that occur during extrusion.

### 3.4. Colour of Extrudates

**Figure 3:**
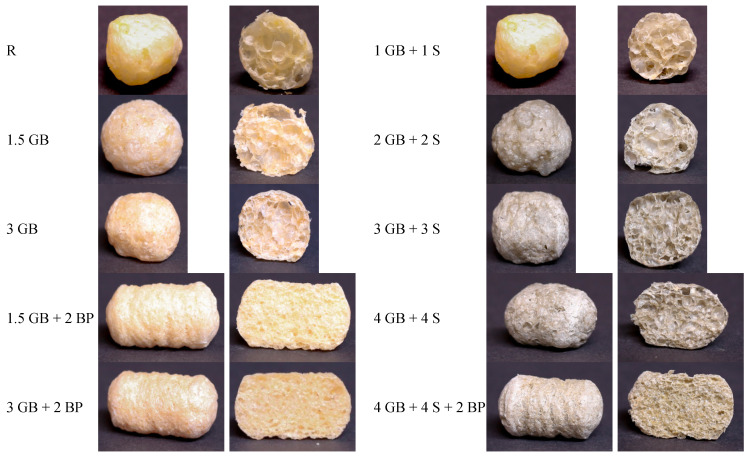
Images of control and enriched extrudates. See Table 2 for explanation of sample codes.

The ingredients used significantly influenced the colour of the obtained extrudates (Table 6). As the amounts of powders increased, the lightness value (L* parameter) decreased, while the green–red colour balance (a* parameter) shifted towards green. Exceptions were observed in extrudates containing cricket powder and baking powder, where a shift in colour balance towards red was noted. Additionally, the addition of both protein sources altered the blue–yellow colour balance (b* parameter), shifting the colour characteristics of the extrudates towards yellow. When comparing the total colour difference (ΔE) values, a significant effect of the two ingredients on the colour parameters of the extrudates was observed. A noticeable differentiation between extrudates can be seen in Figure 3. According to Mokrzycki and Tatol [78], an untrained observer can notice a colour difference between samples if the ΔE value is above 2. The total colour differences of the analysed extrudates ranged from 11.46 (sample 1.5 GB + 2 BP) to as high as 32.13 (sample 4 GB + 4 S), indicating that the colour changes were significant and could be perceived even by an inexperienced observer. The colour of food products is a crucial parameter influencing consumer acceptance. The less processed the product, and thus the closer its colour to the traditionally associated colour, the more willing consumers are to choose it [79,80,81]. Uribe-Wandurraga et al. [68] also observed that the additions of spirulina (0.5−1.5%) significantly increased the greenish tone in the corn extrudates, and total colour difference values of the enriched extrudates were perceptible to the human eye (ΔE in the range of 5−12). In the present study, the protective effect of baking powder on spirulina green pigment retention in extrudates (sample 4 GB + 4 S + 2 BP) was also observed (Figure 3), similar to the findings of a previous study [41].

### 3.5. Sorption Properties of Extrudates

**Figure 4:**
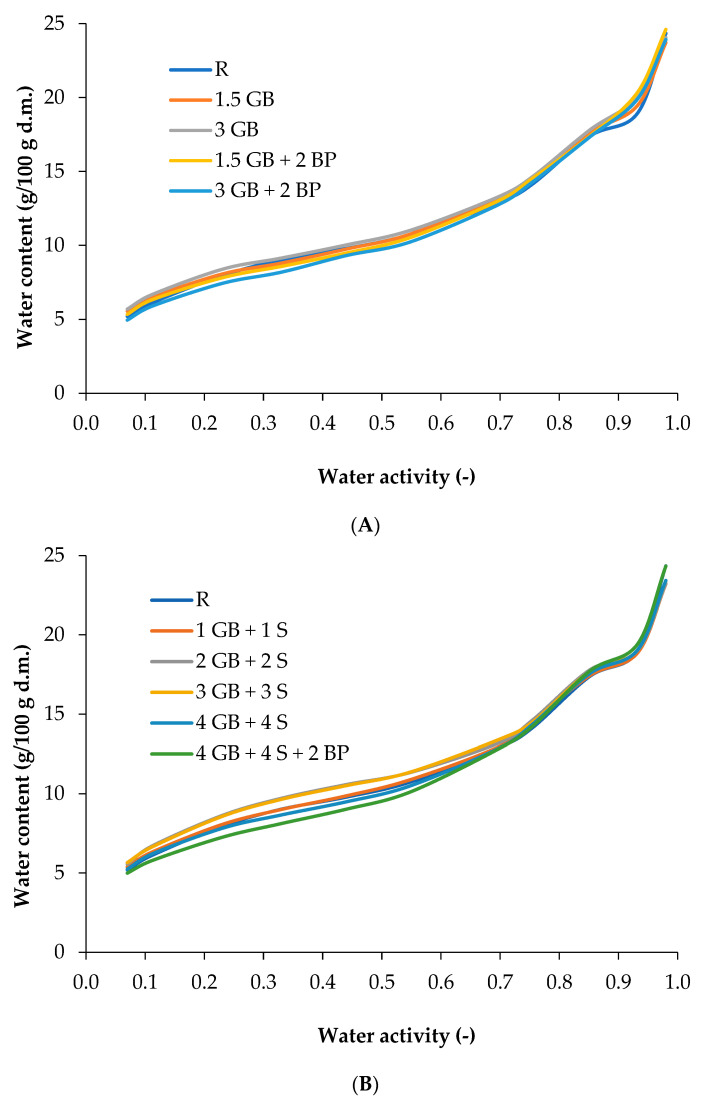
Sorption isotherms of extrudates with field cricket powder (**A**) and with field cricket and spirulina powders (**B**). See Table 2 for explanation of sample codes.

Graphical analysis of the sorption isotherms of the produced extrudates (Figure 4A,B) revealed that the curves were characterised by a sigmoidal shape and continuity over the entire range of ambient water activity (a_w_ = 0.07–0.92). This indicates that there was no change in the structure of the starch–protein matrix of the extrudates, as determined by an increase in the degree of ordering of the individual components. A similar pattern of the sorption isotherm was obtained for corn extrudates enriched with house cricket powder [46] and for extrudates enriched with chlorella and spirulina [42]. The analysis of sorption isotherms provides valuable insights into the behaviour of water in extruded products, showing the relationship between the amount of adsorbed water and the water activity (a_w_) at constant temperature and pressure [82]. Extruded corn snacks are characterised by low water activity, indicating stability and consistent physicochemical and sensory properties during their long-term storage. However, the highly porous structure of extrudates makes them exhibit strong hygroscopic properties [43,83]. Therefore, determining the sorption properties of extruded snacks is crucial to ensuring their quality during their market shelf life [42].

In the water activity range of a_w_ = 0.07–0.23, the desorption process occurred in all extrudates as well as in the control product. However, for extrudates 4 GB + 4 S + 2 BP, the desorption process occurred within a narrower water activity range (a_w_ = 0.07–0.11). Analysing the mutual position of the sorption isotherms revealed differences in the hygroscopicity of the studied extrudates based on the type and amount of ingredients used, specifically cricket and spirulina powders. In region I (a_w_ = 0.07–0.33), the sample 2 GB + 2 S exhibited the highest water content, with an equilibrium water content of 9.71 g/100 g d.m. In sorption region II, which covers the range of multilayer sorption (a_w_ = 0.44–0.69), the sample 3 GB + 3 S had the highest water content, reaching an equilibrium water content of 13.42 g/100 g d.m., closely followed by sample 3 GB with a water content of 13.32 g/100 g d.m. The addition of spirulina slightly increased the hygroscopicity of sample 3 GB + 3 S compared to sample 3 GB. In the third region of the sorption isotherm, covering the range of capillary condensation (a_w_ = 0.75–0.92), extrudates enriched with cricket powder (sample 1.5 GB + 2 BP) had the highest equilibrium water content, while products enriched with cricket powder and spirulina powder (sample 4 GB + 4 S + 2 BP) had the lowest parameters.

In the next stage of the study, the empirical data describing the sorption isotherm were transformed according to the BET model within the range of a_w_ = 0.07−33 to determine the surface structure parameters of the produced extrudates. The BET equation parameters—monolayer capacity (*v_m_*) and energy constant (*c_e_*)—along with the sum of the squares of the deviations of the theoretical values from the empirical ones (SKO) and the values of the standard errors (RMS) are presented in Table 7. The monolayer capacity (*v_m_*) of the GB-enriched extrudates, as determined from the BET model, ranged from 5.41 to 5.95 g H_2_O/100 g d.m. Extrudates enriched with GB and spirulina (S) exhibited a higher range of monomolecular layer (*v_m_*), from 5.66 to 6.87 g/100 g d.m. Thus, it was found that increasing the protein proportion through the addition of spirulina influenced the development of the monomolecular layer capacity. The highest monomolecular layer value among the GB-enriched products was observed in the 3 GB product. Based on the obtained monomolecular layer value (*v_m_*), it was concluded that the addition of 2% BP to the products 1.5 GB and 3 GB contributed to a decrease in the monomolecular layer capacity. A similar effect of the leavening agent was found in extrudates containing spirulina powder (sample 4 GB + 4 S + 2 BP). Among products enriched with GB and S, the highest monomolecular layer capacity was found in sample 2 GB + 2 S. It was concluded that increasing the proportion of GB and S, and thus the higher protein proportion in other extrudates, did not significantly affect the development of the monomolecular layer capacity in samples 3 GB + 3 S, 4 GB + 4 S and 4 GB + 4 S + 2 BP. Based on the evaluation, it was concluded that within the group of extrudates produced, the sample 2 GB + 2 S had the highest shelf life and storage stability due to its highest monomolecular layer value, which significantly protects the product from deterioration by absorbing a certain amount of water. Evaluation of the microstructure of the snack surfaces showed that the addition of spirulina powder to the extrudates increased the sorption-specific surface area (Table 7). The microstructure parameters obtained indicated that the sorption-specific surface area, as derived from the monomolecular layer, was determined by the structural properties of the snack produced during the extrusion process and the interactions between starch and protein particles. The significant development of the monomolecular layer protects the product from quality degradation, as the theoretically determined moisture content corresponding to this layer represents the optimal water content. In this state, the intensity of occurring reactions is reduced, translating to improved storage stability of the produced extrudates [42].

### 3.6. Sensory Properties of Extrudates

To assess the effect of the ingredients used to enrich the extrudates, a sensory evaluation was performed, and the results are presented in Table 8. Although significant colour changes were observed in the instrumental measurements (Table 6), all extrudates enriched with GB alone and the sample 1 GB + 1 S displayed an attractive colour similar to the control extrudates (sample R). The structure changes observed in the tested mechanical properties were reflected in lower appearance scores for the enriched extrudates. Interestingly, the appearance rating for sample 3 GB + 2 BP was not statistically significantly different (*p* > 0.05) from that of the control sample. Enriching food products with new ingredients is a challenge not only due to changes in the production process but also because of the impact these new raw materials have on the sensory properties of the products. To achieve market success, it is insufficient to merely use ingredients that improve the health and nutritional characteristics of products; they must also be acceptable and palatable to consumers [55,84,85]. Numerous literature sources indicate that spirulina has a relatively unattractive aroma, which is often not accepted by consumers in new products [86,87]. Extrudates enriched with spirulina were characterised by significantly lower sensory attractiveness, both in terms of aroma and taste, with scores decreasing as the spirulina content increased in the formulation. However, when evaluating overall attractiveness, it was found that the extruded products with GB up to 3%, along with the addition of BP, as well as product 1 GB + 1 S, were comparably attractive to the control extrudates.

To increase consumer acceptance, it is necessary to develop an appropriate strategy for promoting the use of cricket and spirulina powder in the enrichment of food products, emphasising the nutritional and environmental benefits of these unconventional protein sources. Therefore, the strategy to improve consumer acceptance of products fortified with cricket and spirulina powder should focus on modifying the taste, colour, texture, and crunchiness of the products. Additionally, educating consumers about the nutritional benefits, sustainability aspects, and cultural acceptance of edible insects and spirulina can help change attitudes and increase overall acceptance. From a marketing perspective, organising activities aimed at educating consumers would be advisable. Such activities could include educational or culinary workshops and collaboration with chefs, food bloggers, and influencers to highlight the culinary potential, health benefits, and environmental advantages of using unconventional protein sources like cricket powder and spirulina [88].

### 3.7. Economic Feasibility of Producing Corn Extrudates with Field Cricket and Spirulina Powders

The process of introducing a new product to the market necessitates that manufacturers respond swiftly to intense competition and evolving consumer preferences, with the primary goal of achieving additional profit. Consequently, an essential factor in this context is the economic analysis of the planned venture.

From the manufacturer’s perspective, the implementation cycle of a new enriched extruded product involves taking risks directly related to the expenditures incurred. It can be assumed that initiating the production of extrudates with the addition of cricket powder and spirulina may carry minimal risk, primarily associated with product placement and the potentially prolonged customer acceptance period for new products. For the products analysed, manufacturing costs encompass the cost of raw materials (corn grits, cricket powder, and spirulina powder), electricity consumed in the process, and labour costs.

Using Polish prices obtained from producers and distributors in July 2024 as the basis for the calculation, the purchase cost of 1 kg of corn grits was approximately 6.90 PLN/kg net (1.75 PLN) [89], cricket powder was about 147.70 PLN/kg net (38.00 PLN), and spirulina powder was 80.00 PLN/kg net (20.30 PLN) [90]. Thus, the most significant risk in planning costs may be the fluctuations in raw material purchase prices. Accordingly, the cost components for producing 1 kg of extrudates enriched with 3% cricket powder are the net purchase price of 997 g of corn grits (6.88 PLN) and the net purchase price of 3 g of cricket powder (0.44 PLN). With the addition of 3% cricket powder, the raw material cost of producing enriched corn extrudates increases by 6% compared to producing corn extrudates without additives. Conversely, the cost components for producing 1 kg of extrudates enriched with 4% cricket powder and 4% spirulina powder are the net purchase price of 920 g of corn grits (6.35 PLN), 4 g of cricket powder (0.59 PLN), and 4 g of spirulina powder (0.32 PLN). The addition of 4% cricket powder and 4% spirulina powder results in raw material costs that are only 5% higher than the cost of producing corn extrudates without additives.

Determining the costs directly related to the production process itself, which aims to bring the product to market to achieve the intended profits, is one aspect of the investment. Thus, attention should be paid to the costs associated with sales marketing and logistics. These become particularly important when introducing a new product to the market. The need to familiarise customers with the product, as well as activities to introduce the product into distribution and retail networks, can impose a significant financial burden.

## 4. Conclusions

Based on the study, it was concluded that the produced corn extrudates enriched with field cricket powder and spirulina powder were characterised by favourable nutritional value, including protein content and amino acid profile. The evaluation of sorption properties indicated that the addition of spirulina powder positively impacted storage stability and shelf life by enhancing the development of the monomolecular layer and the sorption-specific surface area. The ingredients used also influenced the physical characteristics of the extrudates. Increasing the amounts of both powders resulted in a reduction in the expansion ratio and an increase in hardness. The enriched extrudates produced were characterised by lower lightness and yellowness compared to the control product, with spirulina powder contributing to green tones. Unfortunately, the addition of both powders negatively affected the sensory acceptability, particularly in terms of colour, aroma, and flavour. Nevertheless, the ratings given by the panellists for each attribute were at least good, except for the product with the highest shares of both protein ingredients, which was rated below 4.0 points. The use of a leavening agent, such as baking powder, generally mitigated the adverse effects on hardness and sensory attributes. 

The results obtained may promote the production of new foods containing edible insects and spirulina. However, producing nutritionally fortified snack products with acceptable physical and sensory properties remains challenging. It is advisable to explore other solutions to improve extrudate quality characteristics, such as fat reduction in insect powder (using defatted meal or insect protein isolate), optimising the cricket powder to spirulina ratio, optimising extrusion parameters (moisture content of the extrusion mix, temperature, screw speed, and pressure), or applying physical leavening agents (supercritical fluid-based extrusion). Future research should also focus on the retention and bioavailability of not only proteins but also other compounds, including bioactive compounds, such as vitamins and antioxidants, sourced from spirulina and edible insects. Additionally, it would be interesting to investigate the possible influence of these additives on the formation of components with adverse effects on the body, such as oxidation products or Maillard reaction products during the extrusion process.

The literature highlights the numerous benefits—health, environmental and economic—of producing and consuming foods and dishes made with insects and spirulina [46,91]. New protein sources, including cricket powder and spirulina, are essential for ensuring food security and represent a goal of sustainable development: feeding a growing population while reducing the negative impact on the environment with a smaller carbon, water, and land footprint compared to traditional protein sources.

When analysing the potential implications of using cricket powder and spirulina in the food industry, the first consideration is the need to increase the nutritional value of enriched products. There is great potential for using unconventional protein sources to enhance the nutritional value of crispbreads, gluten-free baked goods, pasta, confectionery products, energy and protein bars, candy, chips, crackers, and meat analogues. However, before insect- and algae-based products can be preliminarily introduced into the food market, several requirements must be met, including production regulations must be introduced, veterinary education on insect farming (including insect health and welfare) must be expanded, and price premiums for insect farming and algae production (due to high costs) must be implemented. Consumer education should be carried out to increase acceptance of products containing insects and algae. Most importantly, research should be conducted on optimising the composition and improving the sensory properties of products containing unconventional protein sources.

## Figures and Tables

**Table 1:** Chemical composition of raw materials (data based on the manufacturer’s declaration on the unit packaging).

Product	Protein (%)	Carbohydrates (%)	Fibre (%)	Fat (%)
Corn grits	8.3	72.2	2.3	1.7
Field cricket powder	67.0	7.6	1.7	18.0
Spirulina powder	65.0	16.0	3.1	5.4

**Table 2:** Formulations of control and enriched corn extrudates.

Sample	Corn Grits (%)	Field Cricket Powder (GB) (%)	Spirulina Powder (S) (%)	Baking Powder (BP) (%)
R (control)	100.0	0.0	0.0	0.0
1.5 GB	98.5	1.5	0.0	0.0
3 GB	97.0	3.0	0.0	0.0
1.5 GB + 2 BP	96.5	1.5	0.0	2.0
3 GB + 2 BP	95.0	3.0	0.0	2.0
1 GB + 1 S	98.0	1.0	1.0	0.0
2 GB + 2 S	96.0	2.0	2.0	0.0
3 GB + 3 S	94.0	3.0	3.0	0.0
4 GB + 4 S	92.0	4.0	4.0	0.0
4 GB + 4 S + 2 BP	90.0	4.0	4.0	2.0

**Table 3:** Chemical composition of control and enriched extrudates (content of compounds expressed in dry matter).

Sample	Protein (%)	Carbohydrates (%)	Fibre (%)	Fat (%)	Ash (%)
R (control)	8.11 ± 0.06 ^a^	78.17 ± 0.28 ^ce^	0.508 ± 0.04 ^a^	2.83 ± 0.10 ^a^	1.26 ± 0.05 ^a^
1.5 GB	8.53 ± 0.05 ^b^	77.36 ± 0.38 ^b^	0.525 ± 0.02 ^a^	2.98 ± 0.06 ^bc^	1.28 ± 0.05 ^a^
3 GB	8.92 ± 0.10 ^c^	76.29 ± 0.43 ^a^	0.634 ± 0.03 ^b^	2.94 ± 0.13 ^b^	1.45 ± 0.06 ^b^
1.5 GB + 2 BP	8.54 ± 0.03 ^b^	77.33 ± 0.49 ^b^	0.532 ± 0.02 ^a^	3.06 ± 0.11 ^c^	1.30 ± 0.031 ^a^
3 GB + 2 BP	9.03 ± 0.11 ^d^	77.26 ± 0.47 ^b^	0.648 ± 0.02 ^b^	2.92 ± 0.10 ^b^	1.51 ± 0.05 ^c^
1 GB + 1 S	8.89 ± 0.38 ^b^	77.56 ± 0.25 ^e^	0.567 ± 0.03 ^b^	2.99 ± 0.10 ^b^	1.35 ± 0.05 ^b^
2 GB + 2 S	9.36 ± 0.60 ^b^	74.80 ± 0.35 ^cd^	0.625 ± 0.03 ^c^	3.17 ± 0.09 ^c^	1.43 ± 0.05 ^c^
3 GB + 3 S	10.53 ± 0.55 ^c^	74.44 ± 0.94 ^bc^	0.674 ± 0.04 ^d^	3.32 ± 0.09 ^d^	1.48 ± 0.05 ^d^
4 GB + 4 S	11.81 ± 0.40 ^e^	73.81 ± 0.49 ^ab^	0.744 ± 0.03 ^e^	3.52 ± 0.05 ^e^	1.60 ± 0.03 ^e^
4 GB + 4 S + 2 BP	11.23 ± 0.43 ^e^	73.41 ± 0.48 ^a^	0.737 ± 0.02 ^e^	3.47 ± 0.08 ^e^	1.62 ± 0.03 ^e^

Mean values with the same letters in the column were not significantly different (α = 0.05). See Table 2 for explanation of sample codes.

**Table 4:** Amino acid profile and amino acid score (AAS) of control and enriched extrudates compared to FAO/WHO standard for adults.

Sample	Unit	Essential Amino Acids							Non-Essential Amino Acids							
		PHE + TYR	LEU	THR	VAL	ILE	MET + CYS	HIS	ASP	SER	GLU	PRO	GLY	ALA	LYS	ARG
FAO/WHO standard	(mg/g)	41	61	48	25	40	30	23	-	-	-	-	-	-	-	-
R (control)	(mg/g)	5.24 ± 0.09	9.39 ± 0.02	2.14 ± 0.00	2.98 ± 0.01	2.60 ± 0.12	1.51 ± 0.02	1.84 ± 0.00	4.37 ± 0.03	3.08 ± 0.01	12.51 ± 0.27	6.19 ± 0.62	2.06 ± 0.02	7.20 ± 0.04	1.58 ± 0.01	1.97 ± 0.01
	AAS (%)	12.77	15.40	4.46	11.90	6.49	5.04	8.02	-	-	-	-	-	-	-	-
1.5 GB	(mg/g)	6.29 ± 0.20	10.99 ± 0.05	2.43 ± 0.13	3.38 ± 0.07	2.63 ± 0.02	1.96 ± 0.21	1.93 ± 0.01	4.77 ± 0.05	3.69 ± 0.04	16.29 ± 0.25	6.91 ± 0.05	2.47 ± 0.08	5.98 ± 0.20	1.40 ± 0.10	2.64 ± 0.07
	AAS (%)	15.34	18.01	5.07	13.53	6.59	6.53	8.37	-	-	-	-	-	-	-	-
3 GB	(mg/g)	6.65 ± 0.11	11.29 ± 0.17	2.72 ± 0.06	3.85 ± 0.07	3.09 ± 0.04	2.15 ± 0.04	1.98 ± 0.23	5.64 ± 0.10	3.88 ± 0.07	17.01 ± 0.20	7.08 ± 0.02	2.83 ± 0.06	6.57 ± 0.10	1.59 ± 0.29	2.91 ± 0.06
	AAS (%)	16.22	18.51	5.67	15.38	7.73	7.18	8.59	-	-	-	-	-	-	-	-
1.5 GB + 2 BP	(mg/g)	5.24 ± 0.02	9.24 ± 0.05	2.25 ± 0.01	3.22 ± 0.04	2.72 ± 0.01	1.63 ± 0.01	1.71 ± 0.07	4.46 ± 0.01	3.22 ± 0.02	14.08 ± 0.08	5.91 ± 0.10	2.37 ± 0.02	5.32 ± 0.01	0.00 ± 0.00	2.12 ± 0.01
	AAS (%)	12.78	15.14	4.69	12.86	6.79	5.44	7.42	-	-	-	-	-	-	-	-
3 GB + 2 BP	(mg/g)	7.27 ± 0.07	12.29 ± 0.29	2.74 ± 0.04	3.89 ± 0.00	3.27 ± 0.15	2.32 ± 0.02	2.37 ± 0.01	5.50 ± 0.05	4.44 ± 0.07	17.96 ± 0.11	6.72 ± 0.23	2.53 ± 0.03	8.76 ± 0.09	1.78 ± 0.01	2.55 ± 0.05
	AAS (%)	17.73	20.15	5.70	15.54	8.18	7.72	10.28	-	-	-	-	-	-	-	-
1 GB + 1 S	(mg/g)	4.97 ± 0.03	8.83 ± 0.00	2.13 ± 0.01	2.77 ± 0.04	2.63 ± 0.01	1.61 ± 0.02	1.69 ± 0.04	4.43 ± 0.01	2.99 ± 0.14	15.49 ± 0.15	6.32 ± 0.42	2.12 ± 0.03	6.60 ± 0.08	1.79 ± 0.00	2.20 ± 0.03
	AAS (%)	12.13	14.48	4.44	11.06	6.56	5.37	7.35	-	-	-	-	-	-	-	-
2 GB + 2 S	(mg/g)	6.17 ± 0.48	11.96 ± 0.38	2.91 ± 0.10	3.86 ± 0.14	3.67 ± 0.12	1.72 ± 0.19	1.86 ± 0.12	5.36 ± 0.17	4.12 ± 0.13	17.39 ± 0.24	4.60 ± 0.19	2.91 ± 0.08	7.10 ± 0.18	1.64 ± 0.05	2.88 ± 0.12
	AAS (%)	15.04	19.61	6.05	15.43	9.16	5.74	8.07	-	-	-	-	-	-	-	-
3 GB + 3 S	(mg/g)	6.88 ± 0.23	12.05 ± 0.43	3.33 ± 0.03	4.58 ± 0.08	3.79 ± 0.21	1.86 ± 0.10	2.11 ± 0.03	6.74 ± 0.06	4.37 ± 0.06	17.56 ± 0.16	6.76 ± 0.28	3.56 ± 0.05	7.67 ± 0.10	2.71 ± 0.06	3.84 ± 0.06
	AAS (%)	16.78	19.75	6.94	18.31	9.46	6.20	9.17	-	-	-	-	-	-	-	-
4 GB + 4 S	(mg/g)	7.67 ± 0.06	12.40 ± 0.07	3.69 ± 0.01	5.17 ± 0.02	4.75 ± 0.01	2.58 ± 0.15	2.24 ± 0.04	7.58 ± 0.03	4.71 ± 0.02	18.89 ± 0.03	6.97 ± 0.22	4.03 ± 0.01	8.05 ± 0.03	2.73 ± 0.03	4.22 ± 0.08
	AAS (%)	18.72	20.33	7.69	20.67	11.89	8.61	9.74	-	-	-	-	-	-	-	-
4 GB + 4 S + 2 BP	(mg/g)	6.98 ± 0.27	12.16 ± 0.28	3.33 ± 0.29	4.63 ± 0.44	4.07 ± 0.40	2.45 ± 0.16	2.20 ± 0.15	7.10 ± 0.51	4.36 ± 0.33	17.48 ± 1.56	7.77 ± 0.00	3.83 ± 0.28	7.95 ± 0.49	2.83 ± 0.11	3.67 ± 0.21
	AAS (%)	17.03	19.94	6.93	18.52	10.18	8.17	9.56	-	-	-	-	-	-	-	-

See Table 2 for explanation of sample codes.

**Table 5:** Water content, activity, absorption, and solubility of control and enriched extrudates.

Sample	Water Content (g/100 g)	Water Activity (-)	WAI (g/g)	WSI (%)
R (control)	8.39 ± 0.03 ^cd^	0.339 ± 0.011 ^cd^	5.19 ± 0.08 ^a^	7.54 ± 2.06 ^a^
1.5 GB	8.48 ± 0.03 ^d^	0.317 ± 0.003 ^c^	5.23 ± 0.24 ^a^	16.23 ± 1.21 ^b^
3 GB	8.87 ± 0.01 ^e^	0.334 ± 0.003 ^d^	5.29 ± 0.20 ^a^	15.36 ± 0.41 ^b^
1.5 GB + 2 BP	8.27 ± 0.09 ^b^	0.276 ± 0.005 ^b^	5.72 ± 0.09 ^b^	16.67 ± 0.50 ^b^
3 GB + 2 BP	7.86 ± 0.05 ^a^	0.269 ± 0.005 ^a^	5.76 ± 0.24 ^b^	17.90 ± 1.11 ^b^
1 GB + 1 S	8.23 ± 0.06 ^c^	0.354 ± 0.002 ^c^	5.14 ± 0.09 ^a^	20.81 ± 5.06 ^c^
2 GB + 2 S	9.01 ± 0.08 ^e^	0.403 ± 0.016 ^d^	5.28 ± 0.08 ^ab^	15.65 ± 1.70 ^b^
3 GB + 3 S	9.13 ± 0.07 ^f^	0.407 ± 0.015 ^d^	5.38 ± 0.07 ^b^	15.22 ± 1.47 ^b^
4 GB + 4 S	7.83 ± 0.06 ^b^	0.332 ± 0.003 ^b^	5.50 ± 0.17 ^c^	18.01 ± 2.22 ^bc^
4 GB + 4 S + 2 BP	6.93 ± 0.08 ^a^	0.276 ± 0.010 ^a^	5.48 ± 0.09 ^c^	20.61 ± 0.89 ^c^

Mean values with the same letters in the column were not significantly different (α = 0.05). WAI–water absorption index, WSI–water solubility index. See Table 2 for explanation of sample codes.

**Table 6:** Colour parameters (L*a*b* model) and total colour difference (ΔE) of control and enriched extrudates.

Sample	L* (%)	a* (-)	b* (-)	ΔE (-)
R (control)	84.5 ± 0.02 ^df^	−2.94 ± 0.02 ^a^	40.75 ± 0.02 ^e^	-
1.5 GB	75.4 ± 0.06 ^b^	−1.22 ± 0.05 ^b^	31.98 ± 0.11 ^d^	12.78
3 GB	72.9 ± 0.07 ^a^	0.03 ± 0.02 ^d^	28.64 ± 0.05 ^b^	17.00
1.5 GB + 2 BP	78.7 ± 0.07 ^c^	−1.09 ± 0.03 ^c^	31.04 ± 0.08 ^c^	11.46
3 GB + 2 BP	76.2 ± 0.03 ^bc^	0.28 ± 0.02 ^e^	28.52 ± 0.04 ^a^	15.14
1 GB + 1 S	69.3 ± 0.05 ^e^	−4.94 ± 0.03 ^d^	27.47 ± 0.08 ^d^	20.29
2 GB + 2 S	64.7 ± 0.03 ^d^	−4.59 ± 0.04 ^c^	24.41 ± 0.08 ^c^	25.71
3 GB + 3 S	59.2 ± 0.04 ^b^	−4.16 ± 0.04 ^b^	21.88 ± 0.04 ^ab^	31.64
4 GB + 4 S	58.7 ± 0.04 ^a^	−4.12 ± 0.04 ^b^	21.64 ± 0.02 ^a^	32.13
4 GB + 4 S + 2 BP	61.3 ± 0.06 ^c^	−6.20 ± 0.06 ^e^	22.66 ± 0.02 ^b^	29.64

Mean values with the same letters in the column were not significantly different (α = 0.05). See Table 2 for explanation of sample codes.

**Table 7:** BET equation parameters and structure characteristics of control and enriched extrudates.

Sample	Parameters of the BET Equation							Structural Characteristics
	*v_m_*	*c_e_*	R^2^	SKO	RMS (%)	FitstdErr	Fstat	Sorption Specific Surface (m^2^/g)
R (control)	6.01	26.00	0.992	0.99	1.31	0.180	280.59	209.15
1.5 GB	5.73	67.36	0.996	2.16	1.60	0.297	73.64	201.17
3 GB	5.95	59.15	0.995	3.37	2.50	0.317	73.17	209.02
1.5 GB + 2 BP	5.60	63.29	0.996	2.38	2.03	0.282	79.86	196.64
3 GB + 2 BP	5.41	50.05	0.997	2.49	2.55	0.263	92.84	189.97
1 GB + 1 S	6.32	62.40	0.988	0.81	0.58	0.222	164.47	222.01
2 GB + 2 S	6.87	48.92	0.989	1.13	0.72	0.243	178.69	241.37
3 GB + 3 S	6.80	52.93	0.991	1.38	0.83	0.217	211.39	238.86
4 GB + 4 S	6.06	73.60	0.982	4.23	2.54	0.254	110.41	212.90
4 GB + 4 S + 2 BP	5.65	76.80	0.989	3.16	2.84	0.183	178.09	198.65

See Table 2 for explanation of sample codes.

**Table 8:** Sensory characteristics of control and enriched extrudates (number of points).

Sample	Appearance	Colour	Aroma	Taste	Crispness	Overall Score
R (control)	4.86 ± 0.09 ^a^	4.85 ± 0.09 ^a^	4.85 ± 0.09 ^a^	4.97 ± 0.05 ^a^	4.83 ± 0.05 ^a^	4.86 ± 0.05 ^a^
1.5 GB	4.74 ± 0.07 ^b^	4.74 ± 0.07 ^a^	4.74 ± 0.07 ^a^	4.68 ± 0.04 ^b^	4.64 ± 0.06 ^b^	4.65 ± 0.05 ^b^
3 GB	4.63 ± 0.11 ^b^	4.63 ± 0.11 ^ab^	4.63 ± 0.11 ^ab^	4.55 ± 0.08 ^c^	4.45 ± 0.08 ^b^	4.46 ± 0.08 ^b^
1.5 GB + 2 BP	4.77 ± 0.10 ^b^	4.76 ± 0.08 ^a^	4.76 ± 0.08 ^a^	4.76 ± 0.05 ^b^	4.73 ± 0.07 ^a^	4.72 ± 0.07 ^a^
3 GB + 2 BP	4.83 ± 0.07 ^a^	4.82 ± 0.06 ^a^	4.82 ± 0.06 ^a^	4.74 ± 0.08 ^b^	4.75 ± 0.08 ^a^	4.74 ± 0.06 ^a^
1 GB + 1 S	4.77 ± 0.09 ^b^	4.74 ± 0.10 ^a^	4.74 ± 0.10 ^a^	4.64 ± 0.07 ^c^	4.76 ± 0.05 ^a^	4.74 ± 0.06 ^a^
2 GB + 2 S	4.43 ± 0.11 ^bc^	4.46 ± 0.11 ^b^	4.46 ± 0.11 ^b^	4.45 ± 0.10 ^c^	4.57 ± 0.09 ^b^	4.55 ± 0.06 ^b^
3 GB + 3 S	4.24 ± 0.13 ^c^	4.27 ± 0.14 ^b^	4.27 ± 0.14 ^b^	4.25 ± 0.13 ^d^	4.36 ± 0.06 ^b^	4.34 ± 0.07 ^b^
4 GB + 4 S	3.90 ± 0.11 ^c^	3.84 ± 0.09 ^c^	3.84 ± 0.09 ^c^	3.75 ± 0.06 ^e^	3.95 ± 0.07 ^c^	3.85 ± 0.08 ^c^
4 GB + 4 S + 2 BP	4.01 ± 0.15 ^c^	3.95 ± 0.13 ^c^	3.95 ± 0.13 ^bc^	3.84 ± 0.07 ^e^	4.15 ± 0.10 ^c^	4.05 ± 0.06 ^c^

Mean values with the same letters in the column were not significantly different (α = 0.05). See Table 2 for explanation of sample codes.

## Data Availability

The original contributions presented in the study are included in the article; further inquiries can be directed to the corresponding authors.
